# The protein N^α^-terminal acetyltransferase hNaa10p (hArd1) is phosphorylated in HEK293 cells

**DOI:** 10.1186/1756-0500-2-32

**Published:** 2009-03-02

**Authors:** Hiwa Målen, Johan R Lillehaug, Thomas Arnesen

**Affiliations:** 1 Department of Molecular Biology, University of Bergen, N-5020 Bergen, Norway; 2 Section for Microbiology and immunology, Gade Institute, University of Bergen, N-5021 Bergen, Norway; 3 Department of Surgical Sciences, University of Bergen, N-5020 Bergen, Norway; 4 Department of Surgery, Haukeland University Hospital, N-5021 Bergen, Norway

## Abstract

**Background:**

The hNaa10p (hArd1) protein is the catalytic subunit of the human NatA N^α^-terminal acetyltransferase complex. The NatA complex is associated with ribosomes and cotranslationally acetylates human proteins with Ser-, Ala-, Thr-, Val-, and Gly- N-termini after the initial Met- has been removed. In the flexible C-terminal tail of hNaa10p, there are several potential phosphorylation sites that might serve as points of regulation.

**Findings:**

Using 2D-gel electrophoresis and hNaa10p specific antibodies, we have investigated whether hNaa10p is phosphorylated in HEK293 cells. Several differently charged forms of hNaa10p are present in HEK293 cells and treatment with Calf Intestine Alkaline Phophatase (CIAP) strongly suggests that hNaa10p is phosphorylated at multiple sites under various cell culture conditions. A direct or indirect role of GSK-3 kinase in regulating hNaa10p phosphorylation is supported by the observed effects of Wortmannin and LiCl, a GSK-3 activator and inhibitor, respectively.

**Conclusion:**

We demonstrate that hNaa10p protein is phosphorylated in cell culture potentially pointing at phosphorylation as a means of regulating the function of one of the major N^α^-terminal acetyltransferases in human cells.

## Background

Protein N^α^-terminal acetylation is a very common protein modifications in eukaryotic cells, and approximately 80% of soluble human proteins is estimated to carry this modification. In humans, the hNatA complex is the major N^α^-terminal acetyltransferase, acetylating Ser-, Thr-, Val-, Ala-, Gly- N-termini after the initial Met- has been removed by Methionine aminopeptidases [[1], Arnesen T and Van Damme P *et al*., submitted]. The recently described human NatB and NatC complexes are composed of distinct subunits and acetylate different subsets of Met-termini [[3,3], Starheim *et al.*, submitted]. The hNatA complex composed of the catalytic subunit hNaa10p (hArd1) and the auxiliary subunit hNaa15p (hNat1) [4,5] is functionally conserved from yeast displaying almost identical substrate specificity (Arnesen T and Van Damme P *et al*., submitted). Naa10p, Naa11p, Naa15p and Naa16p represent the novel names of Ard1, Ard2, Nat1 and Nat2, respectively, and will be officially presented later this year when the nomenclature of this enzyme class is revised (Polevoda B, Arnesen T and Sherman F, unpublished). hNaa10p and/or hNaa15p has been demonstrated to be important for cell survival [6-8] suggesting important functions for hNaa10p or hNatA mediated acetylation in human cells. In humans, there are two paralogues for each NatA subunit, hNaa10p and hNaa11p [9], and hNaa15p and hNaa16p (Arnesen *et al*., submitted), potentially creating a more flexible and complex system for NatA mediated N-terminal acetylation as compared to lower eukaryotes.

The C-terminus of hNaa10p is flexible [10] and contains many Ser- and Thr- amino acid residues that potentially might serve as a phosphorylation sites that depending on the phosphorylation state may modify the function of the protein. To investigate the possible phosphorylation of hNaa10p and the kinase involved, we analyzed hNaa10p by 2D-gel electrophoresis and Western blotting using anti-hNaa10p. Indeed, the 2-D electrophoresis pattern of hNaa10p indicated multiple isoforms with different charges which is consistent with the hNaa10p protein being phosphorylated at multiple residues. Furthermore, GSK-3 kinase, a highly conserved regulatory serine/threonine protein kinase, is involved in these phosphorylation events.

## Methods

Unless otherwise stated, HEK293 Cells were grown in Dulbecco's modified Eagle's medium (DMEM) supplemented with 10% heat-inactivated foetal bovine serum (FBS) and 2% L-Glutamine in 60 mm dishes (Nunc) at 37°C and 5% CO_2_.

After cell harvesting, the proteins were precipitated by addition of 1 ml cold (4°C) 7% TCA, centrifuged at 13000 *g *for 5 minutes, and the supernatant was poured off. The proteins were resuspended and rinsed in 1 ml cold (4°C) 5% TCA, centrifuged at 13000 rpm for 5 minutes, and the supernatant was poured off again. The protein pellet was rinsed three times in H_2_O-saturated ether (to remove TCA) and centrifuged at 13000 rpm for 5 minutes after each washing step. The pellets were dried at room temperature until they detached from the tube walls and were free of ether, for approximately one hour. Each pellet was dissolved in 100 μl rehydration buffer (6 M urea, 2 M thiourea, 4% (w/v) CHAPS, 20 mM DTT, 0.5% (v/v) Triton X-100, trace amounts of bromophenol blue) and sonicated for 5 × 10 seconds to improve solubilisation. The protein samples were stored at -80°C until analysis by 2D-PAGE. Typically 50 μl of protein sample (from approximately 5 × 10^6 ^cells) was mixed with 300 μl and 200 μl rehydration buffer for 18 and 13 cm IPG strips respectively, and then 1.75 μl ampholytes (0.5%) were added. pH range of the ampholytes varied according to the IPG strips pH-range. The solution was centrifuged at 13000 rpm (Heraeus Biofuge 13), for 10 minutes to separate and remove any insoluble aggregates. The whole volume of the rehydration solution was applied on an Immobiline DryStrip Reswelling Tray (Pharmacia Biotech) with an Immobilized pH Gradient gel DryStrip (IPG strip) (Pharmacia). After rehydration at room temperature for 12–15 hours, isoelectric focusing (IEF) was performed with either Multiphor II™ Flat Bed Electrophoresis Unit (Pharmacia), connected to an EPS 3501 XL Power Supply (Pharmacia), or the Ettan IPGphor™ Isoelectric Focusing Unit (Amersham Biosciences). After isoelectric focusing the IPG strips were put in a Petri dish for direct equilibration by SDS equilibration buffer with 100 mM DTT and thereafter SDS equilibration buffer containing 55 mM iodoacetamide, before analysis by second dimension SDS-PAGE (12.5% polyacrylamide) using the PROTESN II xi 2-D system (20 cm) from Bio-Rad.

SYPRO Ruby protein gel stain (BioRad) was used (according to instruction manual) to visualize proteins in general. To detect the hNaa10p protein, we performed Western blotting of 2D-PAGE gels utilizing the Multiphore II NovaBlot Electrophoretic Transfer Unit. After protein blotting, the nitrocellulose membrane was incubated in 5% dry milk diluted in PBS-Tween overnight at 4°C. The membrane was incubated with anti-hNaa10p antibody [4], diluted (1:500) in 1% dry milk, for 1 hour in room temperature. After washing, 3 × 10 minutes with PBS-Tween, the membrane was incubated for 1 hour with anti-rabbit conjugated to HRP (Amersham Biotech) diluted 1:2000 in PBS-Tween. After washing 3 × 10 minutes with PBS-Tween and once with PBS, ECL technology was used to develop the protein blot (Amersham Pharmacia).

## Results and discussion

In order to investigate potential post-translational modifications of hNaa10p, the protein sequence was analyzed by ELM (ELM) algorithm, publically available at  that predict functional sites in eukaryotic proteins. The algorithm predicted 13 potential phosphorylation sites [11] in hNaa10p sequence recognized by different kinases such as Casein Kinase I (CKI), Casein Kinase II (CKII), and Glycogen Synthase Kinase-3 (GSK-3) (Table 1). In particular, the C-terminal region of hNaa10p had as many as 10 out of the 13 predicted phosphorylation sites. Of note is that the C-terminus (aa 180–235) of hNaa10p has been demonstrated to be unstructured and flexible [10]. Thus, these residues are likely to be accessible for kinases.

**Table 1:** Potential hNaa10p phosphorylation sites predicted by ELM.

*Amino acid position^#^* *(P-site in bold)*	*Peptide Sequence* *(P-site in bold)*	*Predicted Kinase*	Phospho-site pattern*
186–**189**	SPP**S**	CKI	SXX([ST])
213–**216**	SEV**S**		
216–**219**	SET**T**		
228–**231**	SEA**S**		
			
**131**-134	**S**EVE	CKII	([ST])XXE
**189**-192	**S**SGE		
			
180–186 (**182**)	VE**S**KGNS	GSK-3	([ST])XXX [ST]
184–190 (**186**)	GN**S**PPSS		
203–209 (**205**)	ED**S**GGDS		
207–213 (**209**)	GD**S**KDLS		
216–222 (**218**)	SE**T**TEST		
225–231 (**227**)	KD**S**SEAS		
229–235 (**231**)	EA**S**DSAS		
			
149–155 (**152**)	RDL**T**QMA	PIKK	XXX([ST])Q
			
111–117 (**114**)	VRK**S**NRA	PKA	XRX([ST])
			
183–189 (**186**)	KGN**S**PPS	Proline-Directed kinase (e.g. MAPK)	XXX([ST])P

^#^Prediction of phosphorylation sites were performed using bioinformatic algorithm ELM, publically available at . *CKI, CKII and GSK-3 may use acidic or a primed (pre-phosphorylated) residue at the indicated position relative to the phosphorylation site.

The presence of endogenous hNaa10p protein has been demonstrated in several human cell lines, using a specific antibody [4]. 1D-SDS-PAGE and immunoblotting with specific antibody demonstrated the endogenous hNaa10p protein to be approximately 30 kDa as expected from the predicted open reading frame resulting in a protein of 235 amino acid residues.

Due to the ability of 2D-PAGE to separate and visualize a very large number of proteins in complex mixtures of polypeptides according to their p*I *value and molecular weight, it is suitable for the investigation of posttranslational modifications (PTMs) which result in changes in molecular mass and p*I*, such as phosphorylation.

**Figure 1:**
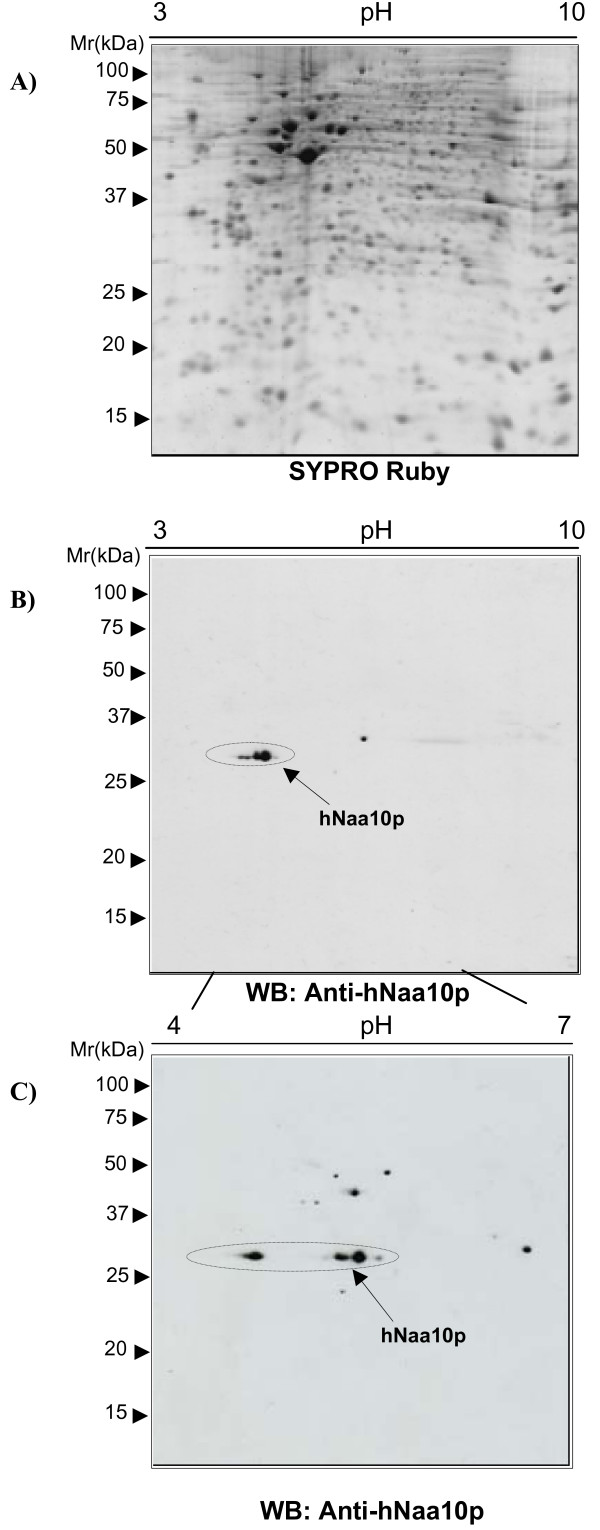
**Detection of the hNaa10p protein by 2D-SDS PAGE**. 80 μg of purified proteins from HEK293 cells were applied to both (A) and (B), and 50 μg proteins to (C). Molecular-size markers are indicated to the left. (A) A 18 cm NL IPG 3–10 was used in the first dimension, followed by SDS-PAGE in the second dimension. Proteins were visualized by using SYPRO-Ruby staining. (B) A 18 cm NL IPG 3–10 was used in the first dimension, followed by SDS-PAGE in the second dimension. Proteins were transferred to a nitrocellulose membrane and immunostained using anti-hNaa10p. (C) A 13 cm IPG 4–7 was used in the first dimension followed by SDS PAGE in the second dimension. Proteins were transferred to a nitrocellulose membrane and immunostained using anti-hNaa10p.

Therefore, this method was applied to study endogenous hNaa10p forms expressed in HEK293 cell cultures. Protein extracts of HEK293 cells were separated by 2D-PAGE and proteins visualized by SYPRO Ruby, clearly demonstrating a nice resolution (Figure 1A). The hNaa10p protein was detected in the 2D-PAGE by protein immunoblot (Figure 1B), and its position corresponded to approximately 30 kDa and a p*I *value of approximately 5.0. The protein was further analyzed by narrowing the immobilized pH gradient used for IEF (Figure 1C). The increased resolution of the first dimension revealed hNaa10p-specific polypeptides with different p*I*s. The "train" of spots observed for the hNaa10p protein could represent multiple isoforms which is typical for multiple posttranslational modifications [12].

**Figure 2:**
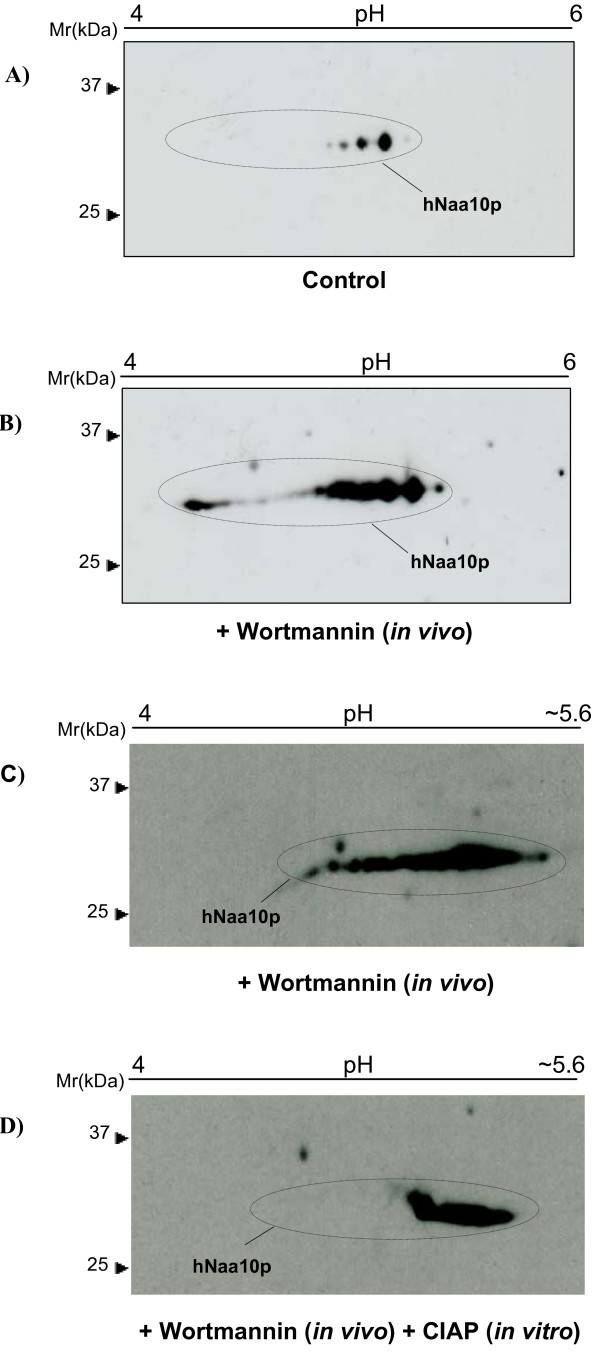
**Activation of GSK-3 kinase leads to a phosphorylation induced acidic shift of hNaa10p**. Cells were *in vivo *stimulated with Wortmannin 1 μM for 8 hours, cells which remained untreated served as control. Furthermore, Wortmannin treated cells were divided in to two aliquots, one was treated with 20 U/ml CIAP enzyme, while the other remained untreated and served as negative control for this experiment. 50 μg of resuspended proteins were analyzed by 2D-SDS PAGE using 13 cm IPG pH 4–7 for IEF, and hNaa10p was detected by immunoblotting using anti-hNaa10p. Molecular size markers are indicated to the left. **(A) **control, unstimulated cells, and **(B) **cells were stimulated with 1 μM/8 hours Wortmannin. **(C) **Cells were stimulated with 1 μM/8 hours Wortmannin *in vivo *(control for D), and **(D) **cells were stimulated with 1 μM/8 hours Wortmannin *in vivo*, and the purified proteins from these cells were treated with CIAP *in vitro*.

The ELM search suggested that hNaa10p might be phosphorylated at 7 residues by GSK-3 (Table 1). We therefore investigated whether inhibition or activation of GSK-3 would result in changes in hNaa10p 2D-PAGE mobility consistent with a change in its phosphorylation state. GSK-3 is a highly conserved and widely expressed serine/threonine protein kinase that plays a central role in the regulation of several physiological processes [13]. Inhibition of phosphatidyl inositol 3 kinases (PI3Ks) by Wortmannin inactivates Akt kinase, which results in the indirect activation of GSK-3 kinase [14]. Thus, addition of Wortmannin should alter the hNaa10p 2D-PAGE mobility pattern if hNaa10p is a GSK-3 substrate. There was an acidic shift of the hNaa10p protein isoforms as a consequence of Wortmannin treatment *in vivo *as compared to control cells (Figure 2A–B), suggesting that hNaa10p is more extensively phosphorylated in the Wortmannin treated cells than in the non-treated cells. The mobility shift corresponded to the addition of 4–6 phosphate groups, estimated using bioinformatic tools . To further investigate the acidic shift caused by Wortmannin treatment, the experiment was combined with *in vitro *treatment with Calf Intestine Alkaline Phosphatase (CIAP). HEK293 cells were treated with Wortmannin; the protein content of the cells was divided into two aliquots, one aliquot was added CIAP, while the other remained untreated serving as control. The results indicate that *in vivo *stimulation of the cells by Wortmannin induces an acidic shift of the protein as expected (Figure 2C), while *in vitro *treatment with CIAP caused reversal of the acidic shift (Figure 2D). This indicates that the acidic shift was mainly caused by phosphorylation induced by GSK-3 activation, suggesting that GSK-3 kinase is involved in the phosphorylation of hNaa10p.

**Figure 3:**
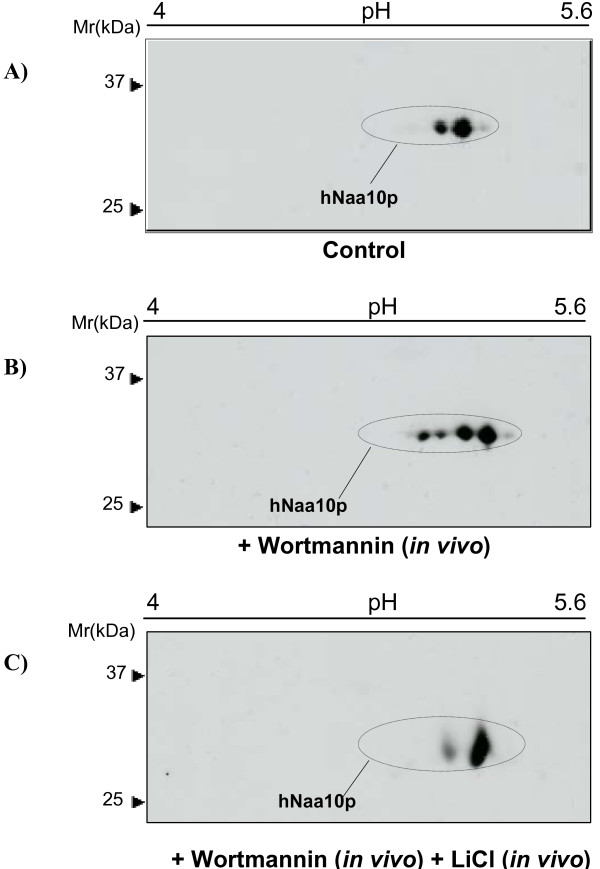
**The phosphorylation of hNaa10p caused by activation of GSK-3 kinase is reversed by a GSK-3 inhibitor**. Cells were stimulated *in vivo *with Wortmannin or both LiCl + Wortmannin. Cells stimulated with neither LiCl nor Wortmannin served as negative control. 50 μg of the resuspended proteins in rehydration buffer were analyzed by 2D-SDS PAGE using 13 cm IPG pH 4–7 for IEF, and hNaa10p was detected by immunoblotting using anti-hNaa10p. The molecular size markers are indicated to the left. **(A) **negative control, cells were unstimulated, **(B) **cells were stimulated by Wortmannin *in vivo *(1 μM/8 hours), and **(C) **cells were stimulated with both Wortmannin (1 μM/8 hours) and LiCl (50 mM) *in vivo*.

Further and to confirm GSK-3 involvement in hNaa10p phosphorylation, we applied *in vivo *treatment of cells with LiCl, an inhibitor of GSK-3 and one of the most effective drugs for the treatment of bipolar (manic-depressive) disorder [13]. To further explore the possibility that hNaa10p protein is phosphorylated by GSK-3 kinase, control cells (Figure 3A) were compared to cells treated with Wortmannin (Figure 3B) or Wortmannin and LiCl in combination (Figure 3C). The Wortmannin treatment results (Figure 3B) confirmed the results presented in Figure 2, that Wortmannin indirectly activates GSK-3 and induces acidic shifts of several hNaa10p isoforms. In this experiment, however, we only observed a Wortmannin-induced mobility shift corresponding to 1–2 phosphorylation sites. Addition of the GSK-3 inhibitor LiCl to cell cultures receiving Wortmannin counteracted the Wortmannin-mediated phosphorylation of hNaa10p (Figure 3C). This result strengthens further that GSK-3 phosphorylates hNaa10p in HEK293 cells.

**Figure 4:**
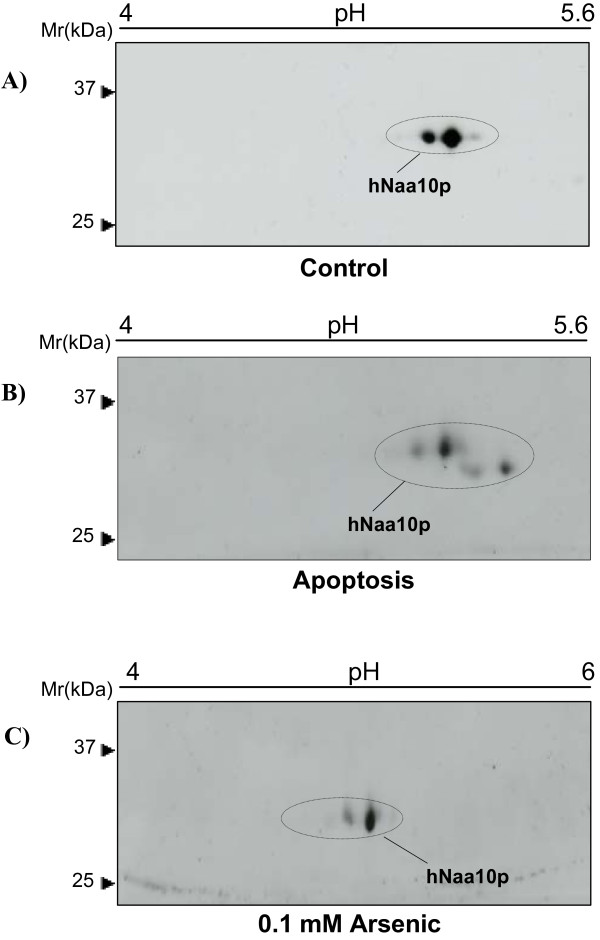
**hNaa10p in apoptotic and arsenic treated cells**. 50 μg protein was analyzed by 2D-SDS PAGE using 13 cm IPG pH 4–7 for IEF. hNaa10p was detected by anti-hNaa10p. The molecular size markers are indicated to the left. **(A) **Control cells harvested after 3 days of culturing, **(B) **apoptotic cells, starved and harvested after 17 days, **(C) **cells were stimulated with 0.1 mM/16 hours arsenic and **(D) **cells were stimulated with 0.3 mM/16 hours arsenic.

We then investigated the phosphorylation status of hNaa10p under different cell culture conditions. To determine the hNaa10p protein state in apoptotic cells, cells were grown to confluence and kept for approximately 15 days without medium change when clear signs of apoptosis could be observed by light microscopy (data not shown). 2D-PAGE-Western blots demonstrated that hNaa10p undergoes cleavage in apoptotic cells (Figure 4B) as reported previously [4]. The cleaved hNaa10p fragment detected by the anti-hNaa10p antibody is more basic than the full-length protein, indicating a cleavage site in the C-terminal region since the amino acid residues in this region are more acidic as compared to residues of the N-terminal region. Interestingly, in the apoptotic cells, hNaa10p is not extensively phosphorylated.

Then, cells were stimulated *in vivo *with arsenic oxide (As_2_O_3_), which acts on cells through a variety of mechanisms, influencing numerous signal transduction pathways and resulting in a vast range of cellular effects that include apoptosis induction, growth inhibition, promotion or inhibition of differentiation, and angiogenesis inhibition [15]. Interestingly, cell stimulation with 0.3 mM arsenic induced an acidic shift of the hNaa10p protein (Figure 4D) while 0.1 mM arsenic did not have any significant effect on hNaa10p (Figure 4C). It should be noted that all arsenic treated cells in these experiments did not show any signs of apoptosis.

**Figure 5:**
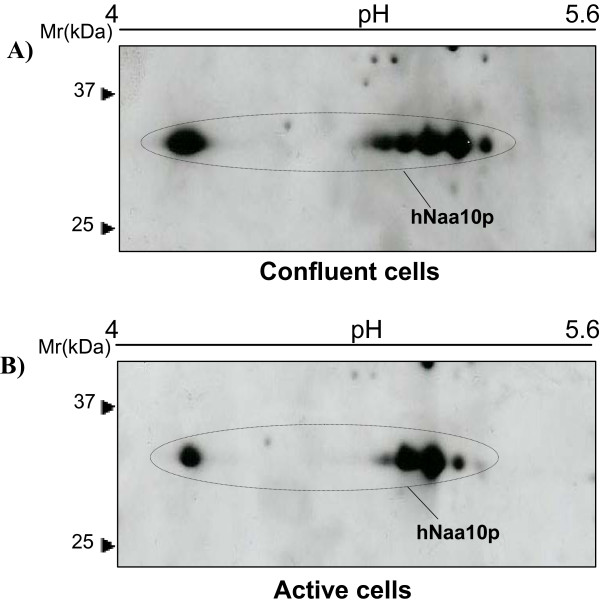
**hNaa10p in confluent and active cells**. 50 μg proteins of each sample resuspended in rehydration buffer and analyzed by 2D-SDS PAGE using 13 cm IPG pH 4–7 for IEF. hNaa10p was detected by immunoblotting using anti-hNaa10p. The molecular size markers are indicated to the left. **(A) **Resting cells were grown for near 6 days without medium change. **(B) **Active cells were splitted into fresh medium 16 hours before harvesting.

hNaa10p was then investigated in HEK293 cells grown to confluence without medium change (for 6 days), these cell cultures are in a steady state with respect to cell number per dish and most cells are presumably in the G0-phase (Figure 5A). These cultures showed no signs of apoptosis as observed by light microscopy. For comparison the hNaa10p status was also analysed in extracts from rapidly dividing HEK293 cells subcultivated with fresh medium 16 hours before harvest (Figure 5B). The results indicate that hNaa10p is more phosphorylated in resting cells than in actively dividing cells.

In conclusion, our results suggest that hNaa10p is phosphorylated *in vivo *and that GSK-3 kinase is phosphorylating and possibly regulating the hNaa10p protein. Since GSK-3 may phosphorylate Ser/Thr-residues if the n+4 position is primed/pre-phosphorylated (Table 1) [16], there are most likely one or more additional kinases responsible for hNaa10p phosphorylation. Further studies are needed to determine whether and which additional kinases are involved in hNaa10p phosphorylation, to reveal the modification sites within hNaa10p and the functional consequences of such phosphorylations.

## Competing interests

The authors declare that they have no competing interests.

## Authors' contributions

HM performed the 2D-gel analyses. TA wrote the manuscript draft. All authors planned the study and participated in manuscript preparation. All authors read and approved the final manuscript.
